# NAP1-Assisted Nucleosome Assembly on DNA Measured in Real Time by Single-Molecule Magnetic Tweezers

**DOI:** 10.1371/journal.pone.0046306

**Published:** 2012-09-25

**Authors:** Rifka Vlijm, Jeremy S. J. Smitshuijzen, Alexandra Lusser, Cees Dekker

**Affiliations:** 1 Department of Bionanoscience, Kavli Institute of Nanoscience, Delft University of Technology, Delft, The Netherlands; 2 Division of Molecular Biology, Innsbruck Medical University, Innsbruck, Austria; National Cancer Institute, United States of America

## Abstract

While many proteins are involved in the assembly and (re)positioning of nucleosomes, the dynamics of protein-assisted nucleosome formation are not well understood. We study NAP1 (nucleosome assembly protein 1) assisted nucleosome formation at the single-molecule level using magnetic tweezers. This method allows to apply a well-defined stretching force and supercoiling density to a single DNA molecule, and to study in real time the change in linking number, stiffness and length of the DNA during nucleosome formation. We observe a decrease in end-to-end length when NAP1 and core histones (CH) are added to the dsDNA. We characterize the formation of complete nucleosomes by measuring the change in linking number of DNA, which is induced by the NAP1-assisted nucleosome assembly, and which does not occur for non-nucleosomal bound histones H3 and H4. By rotating the magnets, the supercoils formed upon nucleosome assembly are removed and the number of assembled nucleosomes can be counted. We find that the compaction of DNA at low force is about 56 nm per assembled nucleosome. The number of compaction steps and associated change in linking number indicate that NAP1-assisted nucleosome assembly is a two-step process.

## Introduction

In eukaryotic cells, DNA is wrapped around histone octamers, forming nucleosomes [1]. As a result, the very long DNA is compacted in order to fit into the nucleus. Perhaps even more importantly, nucleosomes decrease the accessibility of the DNA, as the 146 base pair (bp) linear stretch of DNA wrapped around each histone octamer [2] is inaccessible for proteins that read out the DNA. Indeed, for many important processes like DNA transcription, replication and repair, it is important to understand how DNA is made accessible and inaccessible by the remodeling of the nucleosomes.

In each nucleosome, DNA is wrapped 1.7 times around a disk-like octamer which is built up from eight histones; two copies each of H2A, H2B, H3 and H4, all of which have a positive charge. Histones H2A and H2B interact with each other in a hand-shake-like manner [3] resulting in dimers. Histones H3 and H4 form tetramers in a similar way. The electrostatic force stabilizes the nucleosomes through the negative charge of the DNA backbone and the positive histone charge. At physiological conditions, histones and the DNA do not spontaneously self-assemble into nucleosomes [4], but form large aggregates. It has been found that the necessary order of assembly is first to place the four H3–H4 histones onto the DNA, followed by the two H2A–H2B dimers [4], [5], [6], [7]. In the nucleus, chaperones bring the histones to the DNA in the correct order. Several histone chaperones, such as chromatin assembly factor 1 (CAF1) [8], HIRA [9], and nucleosome assembly protein1 (NAP1) [10] have been characterized as chromatin assembly factors.

Understanding the mechanism of chaperone-mediated assembly is important for many processes in the cell. Binding affinities and rates for the NAP1-mediated histone binding have been reported from biochemical experiments [11], [12]. These bulk assays have, however, the disadvantage that the results are averaged over a population and in time. To obtain insight into the molecular-scale dynamics, we here study nucleosome assembly mediated by the NAP1 chaperone at the single-molecule level. Since in vivo the DNA is under tension and torque, we choose to study the nucleosome assembly with magnetic tweezers which allow full control of the applied force and torque on a single DNA molecule. We measure, in real time, a decrease in the DNA end-to-end length as well as an induced change in supercoiling density upon adding histones and NAP1. We find a correlation between the decrease in DNA end-to-end length and the change in supercoiling density, yielding, on average, a 56±3 nm decrease in length per negative unit change in linking number. To relate our data to results on preassembled nucleosomes [13]–[17], we also perform disruption experiments where we induce nucleosome disassembly by increasing the stretching force on the DNA.

## Materials and Methods

### Magnetic tweezers

**Figure 1:**
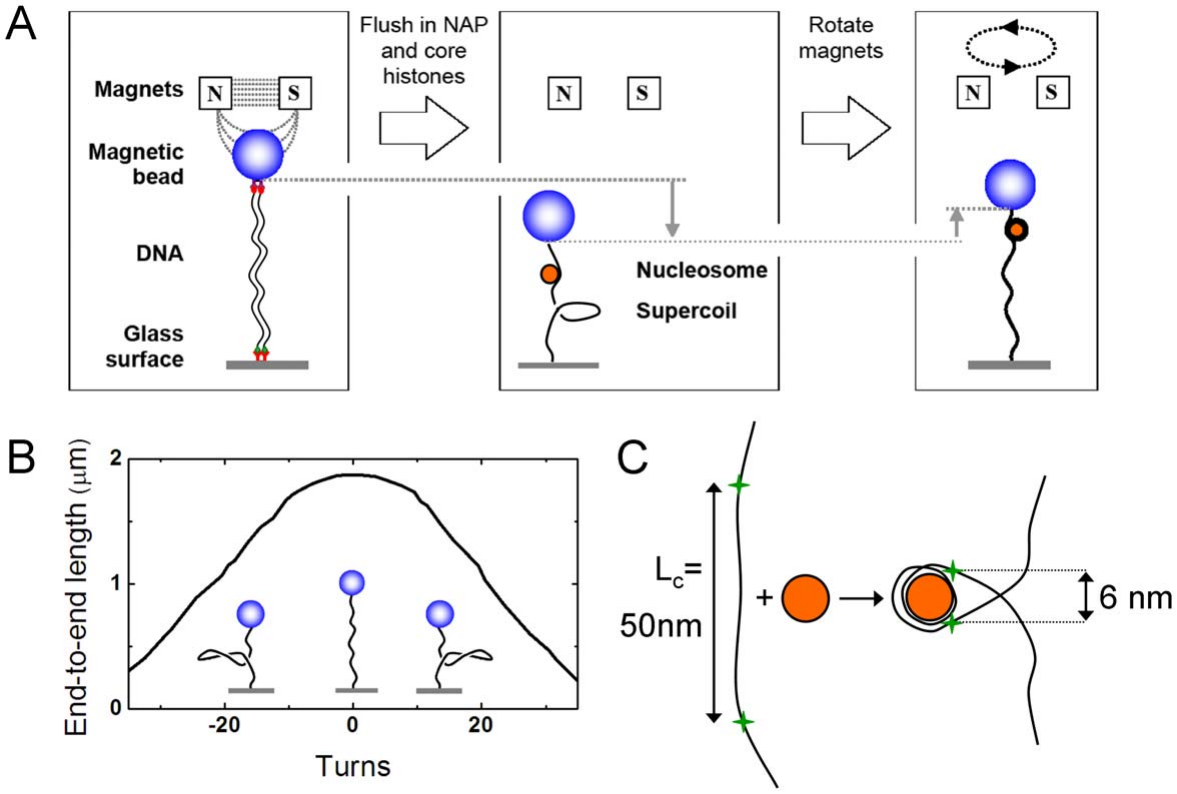
Single-molecule magnetic tweezers (side view in x-z plane). **A** The left diagram shows how an individual double-stranded DNA molecule with DIG labels is tethered to an antiDIG-coated glass surface while at the other end of the DNA, biotin labels attach to a streptavidin-coated magnetic bead. Two magnets above the bead apply a force on the DNA. After flushing in NAP1 preincubated with core histones, nucleosomes are assembled on the DNA, as illustrated in the middle panel. Since nucleosome formation locally winds the DNA around the histone core, positive supercoils are formed in the free DNA because its two ends are torsionally constrained. When the magnets are rotated within the x-y plane, the supercoiling state can be changed as shown in the right panel where the induced supercoils are removed again. **B** Rotation curve at a constant force of 0.3 pN. **C** When a nucleosome is formed from a histone octamer (orange) and DNA (black line), 146 bp (50 nm) of DNA is wrapped in 1.7 turns. The green crosses mark the DNA entry/exit points that are 6 nm apart from each other in the nucleosome. Note that also the finite angle at which the DNA comes out of the nucleosome, which results in a further decrease in end-to-end length.

Single-molecule experiments are carried out using magnetic tweezers [18]. In this assay, one monitors the end-to-end length of a torsionally constrained double-stranded DNA (dsDNA) molecule that is tethered between a surface and a bead to which a force can be applied by external magnets. One end of an 8 kilo base pairs (kb) dsDNA molecule is attached to a glass surface by multiple digoxigenin (DIG)-antidigoxigenin (anti-DIG) bonds. The other end of the DNA has biotin labels and is attached to a 2.8 µm diameter streptavidin-coated magnetic bead (Dynabeads® M-270 Streptavidin) (Figure 1A). The surface is passified by Bovine Serum Albumin (BSA) (New England Biolabs, B9001S), which is incubated in the flow cell for at least one hour at a concentration of 10 mg/ml.

The experiment takes place in a flow cell with an approximate volume of 100 µl. Buffer exchanges occur by placing the new buffer at the inlet of the glass flow cell, and using a syringe pump at the outlet to remove the old solution to a waste container in a smooth and controlled way. This buffer exchange is called ‘flushing’ in the rest of the paper.

**Figure 2:**
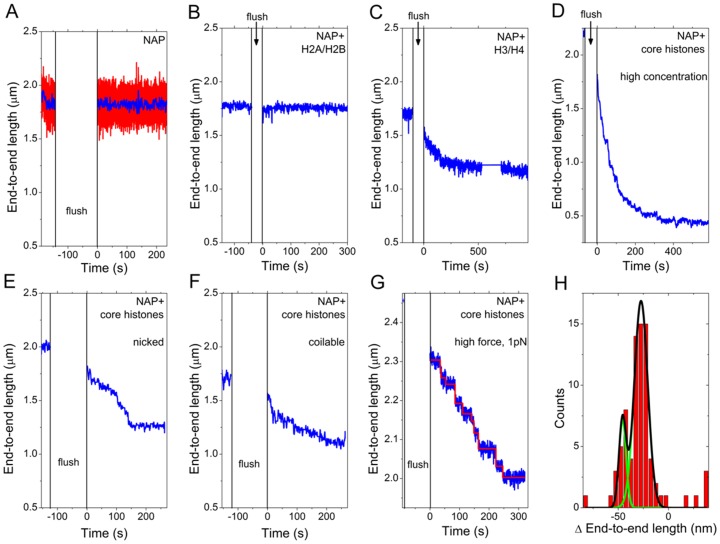
Time traces of the end-to-end length of DNA. In Figures A–E the original DNA end-to-end length before the flush is shown and the force denotes the force applied directly after the flush. During the time when the proteins are flushed in, a higher force (>10 pN) is applied to the bead. Blue lines show the time-averaged data in a moving 100-point average. **A** *NAP1 only:* End-to-end length of a single DNA molecule (height of the magnetic bead) as a function of time. The red line shows raw data at full 100 Hz bandwidth. When a constant 0.3 pN force is applied, the DNA end-to-end length is constant, apart from fluctuations due to Brownian motion. At time t = 0 s the force is again lowered to 0.3 pN, immediately after 3.9 nM NAP1 is flushed in. The end-to-end length of the DNA molecule remains constant after the proteins are flushed in. **B** *NAP1, H2A/H2B:* At t = 0 s the force is lowered to 0.3 pN, immediately after 3.9 nM NAP1 preincubated with 2.6 nM H2A and 2.6 nM H2B is flushed in. The end-to-end length of the DNA molecule is constant, even after the proteins are flushed in. **C** *NAP1, H3/H4:* At time t = 0 s the force is lowered to 0.3 pN, immediately after 5.6 nM NAP1 preincubated with 1.6 nM H3 and 1.6 nM H4 is flushed in. The end-to-end length decreases in an exponential way until a plateau is reached at 66% of the initial length of the DNA. **D** *NAP1, all 4 core histones, high concentration:* At time t = 0 s the force is lowered to 0.7 pN, immediately after 4.8 nM NAP1 preincubated with 12 nM core histones (3 nM of each) is flushed in. The end-to-end length of this coilable molecule decreases instantly in an exponential way until a plateau is reached at 20% of it's original length. **E** *NAP1, all 4 core histones, nicked DNA molecule:* At time t = 0 s the force is lowered to 0.3 pN, immediately after 1.55 nM NAP1 preincubated with 2.1 nM core histones is flushed in. Since this molecule is nicked, no supercoils can be formed. Again, a length decrease is observed. **F** *NAP1, all 4 core histones, coilable DNA molecule:* Same as in E, but in this example the molecule did not have a nick, so supercoils were formed upon nucleosome assembly. **G** *High force assembly:* At time t = 0 s the force is lowered to 1 pN, immediately after 8.6 nM NAP1 preincubated with 11.2 nM core histones is flushed in. Under this higher force of 1 pN, the assembly curve displays individual steps. Blue line shows the tace of the end-to-end length of a nicked molecule; red shows the steps fitted with the step-finder algorithm [28]. **H** *Step histogram:* This experiment in G is repeated for a number of coilable and nicked molecules. They both show a very similar stepping behaviour. The fitted steps of several protein flushes of 5 different molecules are analized with the step-finder algorithm and the result is shown in the histogram. Fitting a Gaussian distribution to the data, two peaks appear (two gaussian fitts in green add up to the total represented by the black line). The most important peak is at −27 nm steps (FWHM = 16 nm); a second smaller peak appears at −46 nm (FWHM = 8 nm).

The position of the bead is tracked using video microscopy at high accuracy (∼5 nm) and speed (frame rate 100 Hz). By placing external magnets close to the bead, a stretching force is applied which can be regulated by the distance between the bead and the magnets. When a constant force is applied, the DNA end-to-end length is constant, apart from fluctuations due to Brownian motion. An example trace is shown in Figure 2A which shows the raw data as well as a moving 100-point average. If the magnets rotate within the x-y plane, the magnetized bead follows this rotary motion and the linking number of the attached dsDNA changes. As a result, positive or negative coils are formed in or removed from the dsDNA. A rotation curve is generated by plotting the end-to-end length of the DNA molecule against the number of applied magnet rotations (Figure 1B). At low force (0.3 pN) the maximum length of the DNA molecule occurs when the supercoil density is zero. At non-zero supercoiling densities, the end-to-end length of the DNA is reduced because plectonemic supercoils are introduced. At any given magnet position, if the magnets are held constant and the DNA is constrained, the overall linking number is conserved (ΔL_k_ = 0). Factors such as protein complex formation can however locally change the linking number. When this occurs in a rotationally constrained dsDNA molecule, the overall linking number is conserved by the formation of supercoils into the DNA. So ΔL_k_ = ΔL_k,nuc_+ΔL_k,DNA_, where ΔL_k_ is the externally applied change by rotating the magnets, ΔL_k,nuc_ is the change in linking number due to nucleosome assembly, and ΔL_k,DNA_ is the change in the linking number of the free DNA which is associated with the formation and removal of supercoils. In other words, when the external magnets are not rotated and the assembly of one nucleosome induces one negative unit of linking number by locally winding the DNA around the histone core, one positive supercoil is formed in the rest of the DNA to keep the overall linking number constant. This is detected as a shift of the peak of the rotation curve at low force. The magnet position of maximum end-to-end length, where no supercoiling is induced in the molecule and thus L_k,DNA_ = 0, now is shifted towards one negative magnet turn to remove the positive supercoil from the DNA, i.e., ΔL_k_ = −1, ΔL_k,nuc_ = −1.0 and ΔL_k,DNA_ = 0.

The appearance of positive supercoils during nucleosome assembly in our tweezer constructs may at first glance seem to conflict the appearance of negative supercoils after nucleosome assembly in bulk experiments. In literature, many bulk biochemical experiments are setup such that during or after nucleosome assembly supercoils are removed by topoisomerase, after which the nucleosomes are removed [19]. The resulting supercoils counted after this procedure are negative. Note, however, that we start with a non-supercoiled DNA construct and we keep it rotationally constrained during the assembly, while after the assembly we measure the supercoiling of the total construct, with the nucleosomes still present. Therefore the supercoils counted by us are positive, as expected for nucleosome assembly.

The slope of the rotation curve in this regime is set by the size of these supercoils: Rotating the magnets one full turn induces one further supercoil and reduces the end-to-end length by the length of DNA absorbed in this supercoil.

### Assembly conditions

In all experiments, we used a buffer consisting of 50 mM KCl, 25 mM Hepes pH 7.6, 0.1 mM Ethylenediaminetetraacetic acid (EDTA), 0.025% Polyethylene Glycol (PEG), 0.025% Polyvinyl Alcohol (PVOH) for crowding, and 0.1 mg/ml BSA for crowding and to prevent nonspecific binding of the histones to the surface. We use a mixture containing recombinant purified *Drosophila* core histones H2A, H2B, H3 and H4 in equal concentrations. Core histones were expressed in *E. coli* Bl21(DE3) Rosetta (Novagen) and purified as described in [20], except that H3/H4 dimers were purified in the same way as H2A/H2B dimers. Expression plasmids were a kind gift of J. Kadonaga. Concentrations of purified histones were determined by SDS PAGE and Coomassie staining and equimolar amounts were combined to obtain octamers. Recombinant *Drosophila* NAP1 was purified according to [21] and preincubated at a concentration of 186 nM with the core histones at a molar ratio of 1∶1.35 for 30 minutes on ice. The incubation buffer contained 50 mM KCl, 25 mM Hepes pH 7.6, 0.1 mM Ethylenediaminetetraacetic acid (EDTA), 0.25% Polyethylene Glycol (PEG), 0.25% Polyvinyl Alcohol (PVOH) and 1 mg/ml BSA. Just before flushing in, the protein concentration is lowered by dilution to 0.9–3.5 nM NAP1 and 1.2–4.7 nM core histones.

### Single-molecule experiments on DNA-nucleosome constructs

We start experiments by characterizing bare dsDNA molecules. For each dsDNA molecule that is tethered between the surface and a magnetic bead, we determine the end-to-end length by measuring the height of the magnetic bead above the glass slide using video microscopy. By repeating this measurement at various applied forces (set by varying the height of the magnets), we can measure the force-distance curve (Figure S1). This curve characterizes the force needed to stretch the DNA to a certain end-to-end length. By fitting a worm-like-chain model to this [22], the persistence length, which characterizes the stiffness of the DNA molecule, is calculated. Experiments are continued if the value is close to the expected 50 nm. With this procedure we prevent considering events where multiple DNA molecules are tethered to the same bead. In addition, a rotation curve is generated at an applied force of 0.3 pN to distinguish between coilable and non-coilable (nicked) molecules. Here, coilable molecules are molecules where supercoils are introduced upon rotation, whereas non-coilable molecules are rotationally unconstrained.

Subsequently, protein complexes are added. During the flushing in of the preincubated protein complexes, the applied force is increased (to 16–20 pN) in order to slow down the assembly reaction [23]–[26] and to prevent sticking of the bead to the bottom of the flow cell. The total flushed volume is five times the volume of the flow cell. Immediately after the flush, the force is lowered again to the value of interest. Video imaging at 50–100 Hz allows to monitor in real time the change in end-to-end length of multiple molecules in the field of view. For a complete dataset the applied force during the assembly experiment was kept low, i.e., below 0.6 pN, to perturb the assembly process as little as possible. Note that at this force, the dsDNA is in its natural B-form configuration and no effects of melting or other forms of DNA have to be taken into account [27].

The change in linking number before and after the experiment is determined by the horizontal shift of the maximum of the rotation curve. Besides a shift, the shape of the rotation curves can also change, i.e. the plateau becomes wider. To determine the shift consistently, we determined the two buckling points, i.e. the points where the peak plateau of the rotation curve begins and ends. The center position between these two points is taken as the center of the rotation curve.

At the end of the assembly experiment, the nucleosomes are disrupted from the DNA at very high (>20 pN) force. Some disruption experiments were carried out at somewhat lower (10–20 pN) forces, with disassembly promoted by the presence of free NAP1 (23 nM) in solution or by higher concentrations of KCl (500 mM), which reduces the binding affinity between the histones and the DNA.

## Results

### Protein-induced length decrease of DNA

We examine the real time NAP1-assisted assembly of core histones onto DNA by continuously monitoring the end-to-end length of single DNA molecules upon addition of histones and NAP1 (Figure 2). The blue traces in 2A–2G are the 1 Hz moving average of the raw data (compare blue and red trace in Fig. 2A). Experiments 2A–C, E–F, are at an applied force of 0.3 pN.

When only NAP1 or NAP1 preincubated with histones H2A and H2B was flushed in, the end-to-end length remained constant, indicating that NAP1 or NAP1 with H2A/H2B does not induce any DNA shortening. By contrast, when NAP1 preincubated with histones H3 and H4 was flushed in, the end-to-end length showed an exponential decrease immediately after (or partly even during) the flushing in of protein (Fig. 2C). After a few minutes, the DNA end-to-end length settled to a new (lower) value.

Similarly, when NAP1 preincubated with all 4 core histones was flushed in, the end-to-end length decreased immediately afterwards (or partly even during the flush). In all experiments the decrease was approximately exponential before the DNA length settled at a new lower value. Example traces for several assembly conditions are shown in Figure 2D–G. The formed DNA-protein complex proved to be very stable: in two-hour measurements, the end-to-end length was constant after reaching the plateau (data not shown).

We observed that the force applied to the DNA affects the speed of assembly. A force increase from 0.3 pN to 1 pN, for example, slowed the assembly down by a factor of about 2 (compare Figures 2E and G; note the different scale on the y-axis in Figure 2G). The speed at which the decrease occurred also depended strongly on the concentration of NAP1 and histones, as illustrated by comparing Figures 2D and 2F for two coilable molecules. Even though the applied force was increased (0.7 pN) in Figure 2D (12 nM core histones), the end-to-end length decreased at a rate of about ∼7 nm/s, notably faster than in Figure 2F (2.1 nM core histones at 0.3 pN) where the decrease occurred at ∼2 nm/s.

We also found that both protein concentration as well as applied force affect the degree of length decrease. For instance, we used concentrations of 0.9–1.6 nM NAP1 and 1.2–2.2 nM core histones (CH) at an applied force of 0.3 pN to obtain a decrease of roughly half the original end-to-end length with an assembly time in the order of 200–300 seconds (Fig. 2E,F). After reaching the lower plateau, we flushed in the same concentration of proteins a second time, which resulted in a further decrease in end-to-end length (data not shown). This indicates that partial adsorption of proteins to the surface occurs.

**Figure 3:**
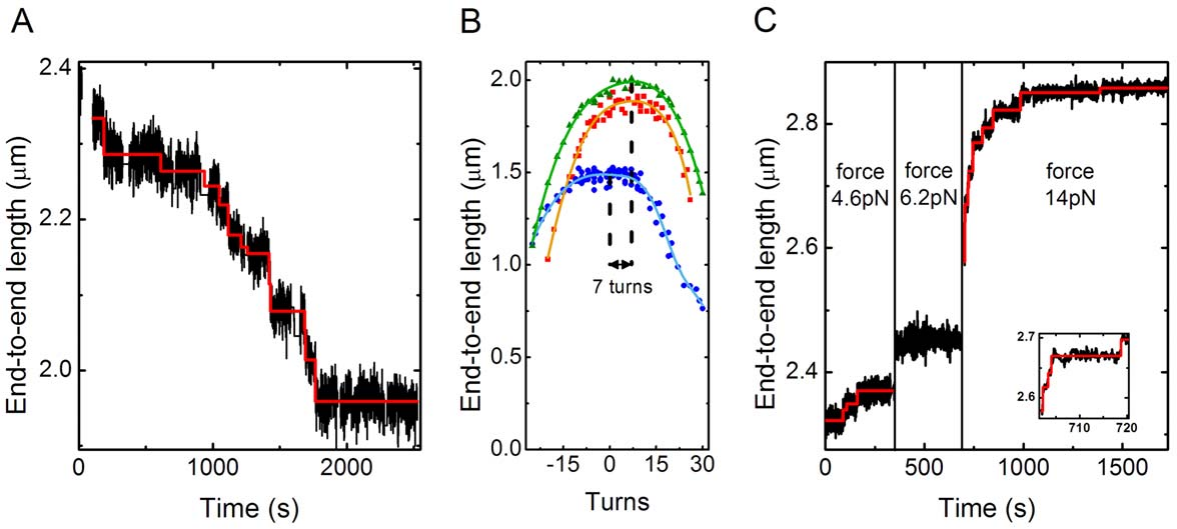
Assembly, change in supercoiling and disassembly on the same molecule. **A** Assembly of NAP1 assisted nucleosomes measured at 1 pN. Data (1 Hz) is shown in black: steps deduced by the step-finder software [28] are shown in red. 12 assembly steps are observed with an average step size of −31 nm. **B** Rotation curve before (red) and after (blue) assembly of nucleosomes. The green curve is measured after pulling at high force. The shift between the red and the blue curve is −7±2. **C** High force disassembly of nucleosomes. The stretching force applied to the molecule is stepwise increased from 3 pN to 17.3 pN, in order to measure both the first and the second disruption event. No steps occurred at the lowest (3 pN) and highest (17.3 pN). A total number of 13 steps is observed, with an average step size of 25±10 nm.

**Figure 4:**
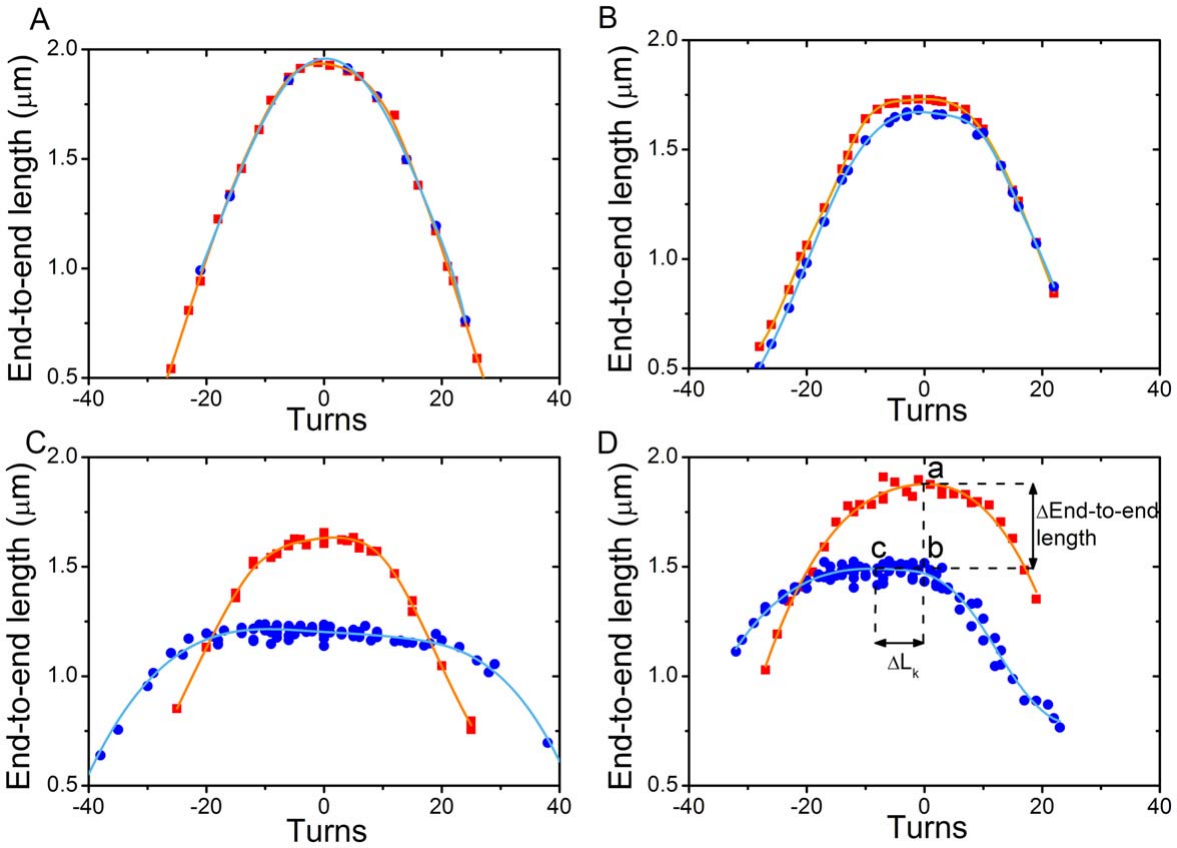
Assembly-induced changes in the rotation curve. **A** *NAP1 addition:* Rotation curves before (red squares) and after (blue circles) a flush of 3.9 nM NAP1 (the experiment from Figure 2A). NAP1 is observed to have no effect on the end-to-end length or on the linking numer. **B** *NAP1, H2AH2B addition:* Rotation curves before (red squares) and after (blue circles) a flush of 3.9 nM NAP1 preincubated with 2.6 nM H2A and 2.6 nM H2B (the experiment from Figure 2B). NAP1 and H2A/H2B have almost no effect on the end-to-end length or on the linking numer. **C** *NAP1, H3/H4 addition:* The rotation curves before (red squares) and after (blue circles) 5.6 nM NAP1 preincubated with 1.6 nM H3 and 1.6 nM H4 is flushed in (experiment as shown in Figure 2C). H3 and H4 do decrease the end-to-end length and broaden the rotation curve, but do not change the linking number (i.e., do not induce a horizontal shift in the position of the rotation curve). **D** *NAP1, all 4 histones:* The rotation curves before (red squares) and after (blue circles) 1.55 nM NAP1 preincubated with 2.1 nM core histones is flushed in (experiment as shown in Figure 2F). NAP1 incubated with all 4 core histones induces positive supercoils and decreases the DNA end-to-end length. At point a, the maximum of the rotation curve before assembly, ΔL_k_ = 0 since magnets are not rotated, ΔL_k,nuc_ = 0 since no nucleosomes are formed yet and ΔL_k,DNA_ = 0 by definition at the peak of the rotation curve. At point c, the maximum of the rotation curve after assembly, ΔL_k_ = −8 since the magnets are rotated 8 turns to bring the construct to the peak in the rotation curve where ΔL_k,DNA_ = 0 by definition, and therefore ΔL_k,nuc_ = −8 since the overall linking number is conserved. At point b thus ΔL_k_ = 0 since the magnets are not rotated, ΔL_k,nuc_ = −8 after nucleosome assembly and ΔL_k,DNA_ = 8 since the overall linking number is conserved. The change in linking number ΔL_k,nuc_ due to nucleosome formation can thus be measured by measuring the number of the turns by which the maximum of the rotation curve is shifted to the left. The decrease in end-to-end length during assembly is caused by nucleosome assembly (see Figure 1C) as well as by the change in supercoiling state. The end-to-end length change introduced by nucleosome assembly can be independently read off from the difference in height of the peak of the rotation curve.

When measurements were performed at higher force (1 pN), it was possible to distinguish individual assembly steps. At these higher forces, the signal-to-noise ratio increases due to a reduced Brownian motion and a decreased assembly rate. The traces can therefore be analyzed using the step-finder algorithm developed by Kerssemakers et al. [28], which finds steps hidden in Gaussian noise without bias towards size or duration. Figure 2G shows an example of stepwise assembly at 1 pN as well as a fit of the steps. The molecule shown here was not rotationally constrained, so no supercoils were formed and the steps are merely due to shortening of the DNA by local wrapping of DNA around histones. We performed this type of experiments for both coilable and non-coilable (example in Fig. 3A) molecules, and both are found to exhibit a similar stepping behavior. When analyzing several molecules, we found an average step size of about −27±8 nm with some steps that are approximately twice as big, (see Fig. 2H). Note that a difference between coilable and noncoilable molecules, e.g. an expected extra decrease due to the formation of supercoils, was not readily observed in these experiments when starting at supercoiling densities of 0 and lower. As discussed in the next section, this is due to a significant broadening of the plateau of the rotation curve when nucleosomes are being assembled (Fig. 4D).

Since the partial nucleosome assembly with NAP1 and only histones H3 and H4 also induced an end-to-end length decrease, we investigated the step size of this process as well. It turned out that the stepping behavior was similar to the assembly of NAP1 with all four core histones (Figure S2), with a most probable step size of about −25 nm.

### Supercoiling state of the DNA-protein construct

The decrease in end-to-end length is strongly indicative of nucleosome assembly. As shown in our experiments of NAP1 preincubated with histones H3 and H4 (Fig. 2C), however, a (stepwise) decrease in length alone, does not rigorously prove nucleosome assembly. An important additional experimental parameter is therefore the induced change in the number of supercoils in the DNA.

Figure 4 shows the rotation curves before (red squares) and after (blue circles) the assembly experiments. The rotation curve obtained after flushing in a buffer with only NAP1 perfectly overlaps the rotation curve measured before the flush (Fig. 4A). We therefore conclude that the NAP1 protein does not affect the mechanical and structural characteristics of the DNA molecule. Furthermore, when NAP1 was preincubated with only the H2A–H2B dimer, the end-to-end length as well as the rotation curves also remained largely unchanged (Fig. 4B). Addition of NAP1 preincubated with H3–H4 to DNA did, however, have a significant effect on the end-to-end length (Fig. 2C). In this case, the real time end-to-end length decrease was found to be comparable to data obtained upon adding NAP1 with all four histones (Fig. 2E–F). Importantly, however, while the rotation curve broadened and the maximum of the peak was lower than before, the center of the peak did *not* show a shift towards positive or negative supercoiling (Fig. 4C).

In all our assembly experiments with NAP1 preincubated with all four histones on topologically constrained (coilable) DNA molecules, we observed that positive supercoiling was induced in the non-histone-bound free DNA upon addition of the histones and NAP1. A typical example is shown in Figure 4D. The peak of the curve after the assembly is clearly shifted to the left and is less high than the peak of the curve measured before.

**Figure 5:**
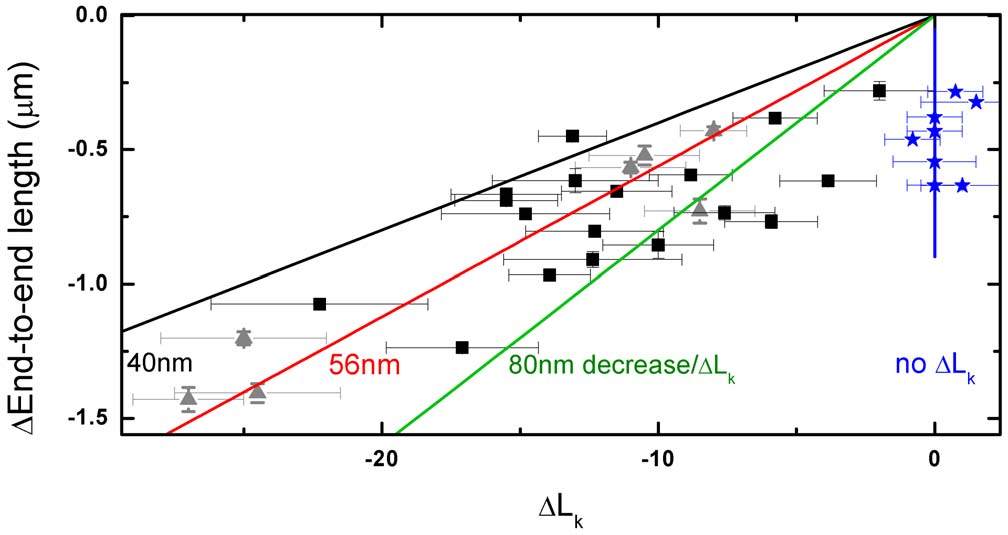
The difference in end-to-end length of the peak of the rotation curves before and after the assembly experiment, plotted against the change in supercoiling state as deduced from the shift of the maximum of the rotation curve (ΔL_k,nuc_). Black squares show the results for experiments of NAP1 preincubated with all four histones assembled at 0.3 pN, grey triangles at 1 pN. Blue stars are the results from experiment of NAP1 preincubated with histones H3 and H4 only. A linear fit through the origin of the black and grey data (red line) reveals a slope of 56±3 nm per turn (red line). If one assumes that the assembly of one nucleosome results in the formation of 1 positive supercoil, this means that the assembly of one nucleosome decreases the end-to-end length by 56 nm at 0.3 pN. For comparison, lines with a slope of 40 (black) and 80 (green) nm decrease per negative unit change in linking number ΔL_k,nuc_ are shown. The experiments with H3 and H4 only do show a decrease in end-to-end length but do not show a change in linking number.

Next to the change in supercoiling upon addition of the histones and NAP1, we observed a concomitant decrease in DNA length and a broadening of the rotation curve. To study the correlation between the decrease in end-to-end length and the change in linking number, these two parameters were plotted against each other in Figure 5. The black and grey data points indicate the change in linking number and end-to-end length of coilable molecules after flushing in NAP1 preincubated with all four histones. The applied force during assembly was 0.3 pN for the black squares and 1 pN for the grey triangles. The blue stars represent the data from experiments with NAP1 preincubated with only histones H3 and H4. The latter shows no change in linking number. As expected, for the data on NAP1 preincubated with all four histones, larger length decreases correlate with larger changes in linking number. A roughly linear interdependence was observed, with a same trend for data acquired at 0.3 pN and 1 pN. The slope indicates the change in length per linking number. The average decrease in end-to-end length per unit change in linking number ΔL_k,nuc_ is 56±3 nm (the error is the standard error of the fit). The variation in the data is appreciable. Indeed, since the rotation curve becomes relatively broad, the error in determining the peak maximum is relatively large. For comparison, lines with a slope of 40 (black) and 80 (green) nm decrease per unit ΔL_k,nuc_ are shown.

These data show that a (stepwise) decrease in end-to-end length alone does not prove nucleosome formation. A decrease in end-to-end length combined with a change in supercoiling density, however, is strong evidence of NAP1-assisted assembly of complete nucleosomes.

### Force-induced disassembly

**Figure 6:**
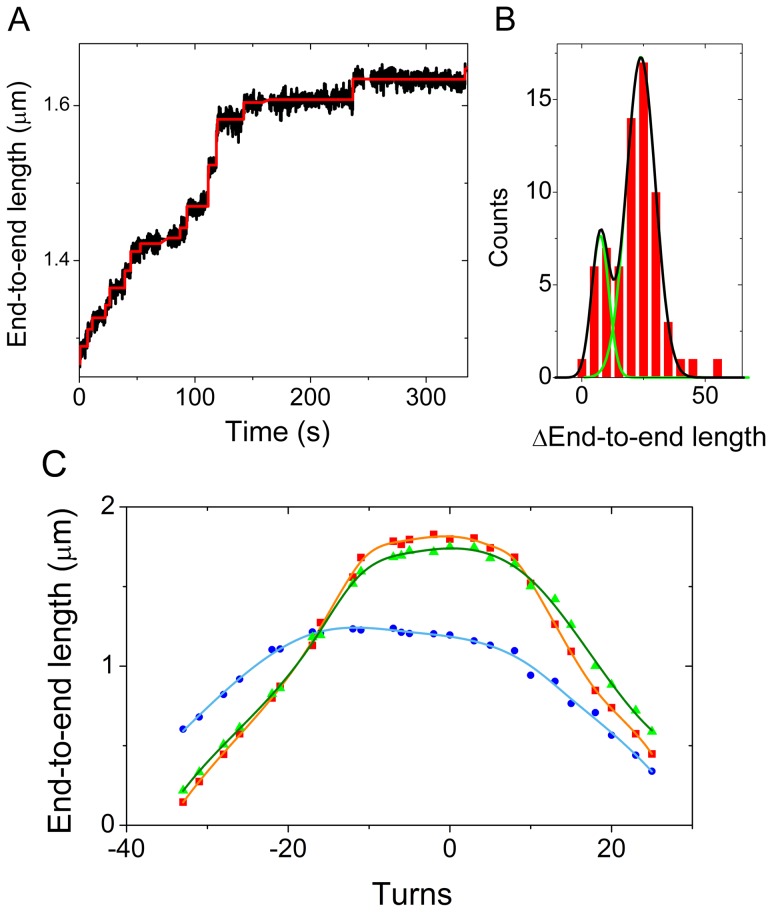
Force-induced disassembly of nucleosomes from DNA after assembly with NAP1. **A** Upon increasing the force to 14 pN at time t = 0, the end-to-end length increases stepwise due to nucleosome unwrapping. Raw data are shown in black, the steps calculated by the step-finder software [28] are shown in red. **B** Histogram of the force-induced disassembly steps of 5 molecules. Two Gaussian peaks are fitted to the histogram (and these two peaks in green add up to the total represented by the black line). The largest peak is observed at 24 nm (FWHM = 14 nm). A smaller second peak is seen at 7.5 nm (FWHM = 8 nm). **C** Rotation curves before (red squares) and after (blue circles) a NAP1-assisted nucleosome assembly experiment. After applying high pulling forces, a third rotation curves is measured (green triangles) that is close to that of the DNA before assembly of nucleosomes, showing that one can recover the nonsupercoiled state of the original bare DNA.

After the assembly of nucleosomes, we applied a high force of up to more than 20 pN to disrupt them again. This resulted in an immediate length increase due to the stretching of the free DNA, but we also observed a subsequent stepwise increase in length which can be attributed to force-induced disassembly of nucleosomes. An example trace of disassembly at a constant force of 14 pN is shown in Figure 6A. The black data is the 1 Hz moving time average of the raw data. Using the step-finder algorithm [28], we fit the number of steps as well as the step size. By analyzing multiple molecules we found that steps were 24±7 nm (Fig. 6B). Upon applying forces well above 20 pN, nearly all the nucleosomes were disrupted and the original bare DNA molecule was recovered. This was judged by the similar end-to-end length and supercoil state before the assembly experiment and after the disassembly experiment (Figure 6C). Instead of applying high forces (>20 pN) it was also possible to recover the original bare DNA by using high salt concentrations (500 mM KCl added to the buffer) or an excess of free NAP1 protein in solution (25 nM) that competes for histone binding. The combination of assembly-disassembly experiments provides reliable information on the number of assembled nucleosomes. Moreover, this experimental setup is suitable to perform multiple consecutive assemblies-disassemblies on the same molecule, which is an important advantage over other single-molecule experiments on nucleosome (dis)assembly [13]–[15].

## Discussion

### DNA-wrapping-induced end-to-end length decrease

Our experiments on NAP1 as a histone chaperone reveal that in vitro nucleosome assembly can be monitored in real time at the single-molecule level. The most obvious evidence for that is given by the step-wise decrease in DNA end-to-end length with step sizes of −27±8 nm at 1 pN force (Fig. 2H). However, steps occurring during assembly do not rigorously prove that nucleosomes are formed, since assembly of NAP1 with only histones H3 and H4 yielded a similar length decrease in a stepwise manner. The step size for rotationally constrained molecules could be a combination of DNA wrapping and supercoil formation. To separate these two contributions the maximum of the rotation curve can be determined, which reveals an independent estimate of those parameters. The difference in height of the rotation curve peaks before and after the experiment is a measure of the length difference due to the wrapping of DNA around histones (Fig. 1C). Next to direct DNA length decrease due to wrapping and supercoil formation, also the angle at which the DNA leaves the nucleosome adds to the decrease in end-to-end length of the DNA in the magnetic tweezers.

In our assembly experiments we measure a 56±3 nm decrease in end-to-end length per formed unit change in ΔL_k,nuc_. Within errors, this is in agreement with a previously reported value of 55 nm [15] from measurements of the change in supercoil state due to disruption of preassembled regular fibers. Assuming a one-to-one relation between the unit change in ΔL_k,nuc_ and the number of formed nucleosomes (see discussion in the next paragraph), one might expect that our measured decrease in end to end length will be comparable to the length increase in high-force disruption experiments. It has previously been reported [16] that disassembly occurs in two separate steps: a first step occurring at about 3 pN with a step size of around 21 nm, and a second step with a size of about 22 nm starting from 8–9 pN. Adopting these numbers, one would for our low-force (all rotation curves are measured at 0.3 pN) conditions naïvely predict a decrease in end-to-end length of 31 nm per formed nucleosome, since the end-to-end length at 0.3 pN is 73% from that at 3 pN (see the force-distance curve in Figure S1), and 70% from that at 8.5 pN, yielding 0.73×21 nm+0.70×22 nm = 31 nm. Clearly this value is lower than the 56 nm number observed in our assembly experiments. It is important to note however that the calculation does not consider effects that may take place in the low-force regime, such as local bending of the DNA at the nucleosome site, nucleosome-nucleosome interactions, and the fact that the end-to-end length will also decrease due to a lowering of the persistence length of the DNA molecule upon nucleosome formation [29]. We believe our directly measured value of the NAP1-assisted nucleosome assembly to be the most reliable estimate for the DNA shortening upon nucleosome assembly.

### Change in linking number

To measure the number of formed nucleosomes in rotationally constrained molecules, the change in supercoil density can be studied in addition to the wrapping-induced end-to-end length decrease. In all our assembly-experiments we observe a change in the linking number: the peak of the rotation curve, i.e. the position at which no supercoils occur, is shifted towards negative turns, indicating that positive supercoils are induced in the free DNA by the NAP1-assisted nucleosome formation.

It has been reported that the formation of one nucleosome changes the linking number (ΔL_k,nuc_) by −1.0 [30]–[32]. Assuming such a one-to-one relation between the change in supercoiling and the number of formed nucleosomes, one can conclude that the formation of one nucleosome is associated with an end-to-end length decrease of about 56 nm.

While the measured relation between end-to-end length decrease and change in supercoiling density shows a general trend comparable with previously reported data on preassembled fibers, some data points in Figure 5 display a somewhat larger decrease in end-to-end length (black and grey data points on the right). Based on our control experiments with NAP1 and H3–H4, which show a decrease in end-to-end length but no change in supercoiling density, this suggests that nucleosomes can be assembled completely but also partially. Determining the number of fully assembled nucleosomes based on the change in supercoiling density is therefore more sensitive and reliable than an estimate based on a mere change in end-to-end length. Based on the supercoil formation, the number of assembled nucleosomes on our 8 kb DNA was found to range between 4 and 27, depending on our choice of protein concentration and assembly force.

In ref. [26] Gupta et al. used magnetic tweezers to study the yNAP1-assisted assembly of nucleosomes on rotationally constrained and nicked DNA molecules. They concluded that rotationally constrained DNA has a slower assembly rate and a lower number of assembled nucleosomes as result of the build up of torsional stress. In our experiments, however, where we simultaneously measured both nicked and coilable molecules. We found that both types of molecules showed comparable assembly curves and coilable molecules also assembled quite a large number of nucleosomes. Importantly, we do full rotation curves after the assembly with NAP and core histones. Indeed, our results with the assembly of histones H3 and H4 with NAP1 show that a (stepwise) decrease in end-to-end length alone is insufficient to rigorously prove nucleosome assembly. Partial assembly can also decrease the end-to-end length as well as broaden the rotation curve (as discussed in the next section). Measurement of the change in linking number appears necessary to be conclusive. Such data also provide insight into the ratio between complete nucleosomes and partial nucleosomes.

It is furthermore useful to discuss our findings in relation to the experiments reported by Torigoe et al. in ref. [33]. They assembled proteins in the presence of topoisomerase I on closed circular DNA templates. After the assembly all proteins are removed and the extent of supercoiling is measured. In their assembly of all four core histones with NAP1 they detected only small amounts of supercoiling, while we see a clear effect of the assembly on the linking number of our constrained molecules. This difference can be explained by the conditions of assembly: Their construct effectively does not induce any torsional stress during assembly due to the topoisomerases. The absence of torsional stress could effect the assembly efficiency and/or the configuration of the histones onto the DNA. Indeed, ref [34] describes an example of different nucleosomal configurations depending on the torsional stress.

### Broadening of the rotation curve

Although evidence from some bulk experiments pointed to a ΔL_k,nuc_ = −1.0 per formed nucleosome, other work suggests that the value of ΔL_k,nuc_ = −1.0 is merely an average and that the nucleosome can be in different conformations. It has been proposed that ΔL_k,nuc_ can vary between −1.4 and −0.4 [15], [35], [36], depending on the crossing angle of the entry/exit DNA. *In vivo*, a mixture of nucleosome conformations may occur in chromatin. A change in supercoil density or other conditions, such as salt concentration, could shift one configuration to another, thereby changing the relative occurrences. A distribution in ΔL_k,nuc_ should not have an effect on the overall shift of the rotation curve, since at the peak position we compare the situations without torque before and after the assembly.

The shape of the rotation curve on the other hand is expected to change. Starting from the peak where ΔL_k,nuc_ = −1.0, negative rotations will drive more and more nucleosomes into the configuration where ΔL_k,nuc_ = −1.4 per nucleosome, whereas positive magnet rotations will result in more nucleosomes in the configuration where ΔL_k,nuc_ = −0.4 per nucleosome. The broad plateau of the rotation curves measured after nucleosome assembly may represent this spread in ΔL_k,nuc_. The nucleosome thus may potentially absorb a ΔL_k,nuc_ of −0.4/0.6 upon changing the applied torsion. If the energy needed to change the nucleosomal conformation is smaller than the energy needed to supercoil the dsDNA, these two processes are separated, resulting in a plateau, where torsion is absorbed in changing nucleosome conformations, at the top of the rotation curve with edges, where supercoiles are formed. In this picture, the width of the plateau also gives an indication of the number of nucleosomes, since one nucleosome broadens the plateau by 1 (i.e. the range from −1.4 to −0.4).

Besides a broadening of the rotation curve after nucleosome assembly, we also measure a broadening after flushing in NAP1 with only histones H3 and H4. In this case, the center of the rotation curve does not shift, meaning that the overall linking number is conserved. But a clear broading is observed. To understand the underlying cause of this, it is useful to consider the possible structures that may be formed. In ref. [37] it was found that NAP1 with histones H3 and H4 most likely adopt a tetrasomal configuration related to that observed in the nucleosome. Other studies have shown that the tetramer is more heterogeneous on its own than when sequestered in the octamer [38]. Refs. [39], [40] propose that the two H3–H4 dimers can rotate in the reverse direction around their H3-H3 interface, possibly flipping the tetramer from a left- to a right-handed superhelical form under positive torsional stress. The tetramer in this case absorbs both positive and negative torsional stress before the bare DNA forms supercoils, which could explain our result of broadening in the rotation curve after H3 and H4 assembly.

### Nucleosome disassembly

Upon high-force disruption of nucleosomes, we obtained a step size of around 24±7 nm for an increase in end-to-end length. This is in line with reported values from unwrapping experiments where it was reported that approximately 21 nm of DNA is released upon initial looping off of the DNA from the histone core at forces of 3 to 7 pN, followed by a second irreversible unwrapping event that occurs between 5–25 pN, where a similar amount of 22–25 nm of DNA is released [13], [14], [17], [41]. In our experiments the total number of detected steps divided by two thus should equal the number of assembled nucleosomes. For the example molecule shown in Figure 6A, the 15 steps thus correspond to 7 to 8 nucleosomes.

### Two-step assembly

In the real-time measurements of NAP1 assisted assembly of all four histones, we measured a stepwise decrease in end-to-end length. Since we found that the total number of assembly steps was twice the unit change in linking number, we envision that NAP1 assembles complete nucleosomes in two separate steps. We propose the first NAP1-assisted assembly step to involve formation of a nucleosomal substructure composed of histones H3 and H4, which does not induce a change in linking number. This is supported by the results shown in Figure 2B and 4B, which show that NAP1 with H2A and H2B do not change the DNA end-to-end length, nor the linking number. In contrast, the results in Figures 2C and 4C showed that histones H3 and H4 do have an effect on the DNA end-to-end length but not on the linking number. The step sizes of NAP1-assisted assembly of H3 and H4 are comparable to that of NAP1 with all four histones. The exact structural configuration formed by the proteins cannot be concluded from our assay. As various papers, for example ref. [37] have suggested that NAP1 assembles H3 and H4 in the tetrameric conformation, it is most likely that also in our experiment the histones associate as a tetramer with the DNA and the DNA folds onto the complex. In a second step, NAP1 brings the histones H2A and H2B to this structure. Now a complete octamer is formed and the DNA completes the 1.7 turns around the octamer, forming a complete nucleosome and changing the linking number by −1.0. The two-step assembly process we suggest is comparable with the ‘nascent’ and ‘mature’ chromatin as described in ref. [33], with the important difference that our data do show that NAP1 alone is able to assemble complete nucleosomes with a change in linking number of the rotationally constrained DNA, instead of only ‘nascent’ chromatin.

We can verify this two-step assembly hypothesis by checking the consistency in three parameters measured in our experiment. One is the number of steps measured during nucleosome assembly. The second is the change in linking number upon nucleosome assembly. The third one is the number of steps measured during disruption of the nucleosomes. In Figure 3 we show data for these three parameters collected in one experiment on the same molecule. These separately measured data should all give the same estimate of assembled nucleosomes, based on the two-step assembly theory.

The assembly-experiment of Figure 3A, done at 1 pN, revealed a step-wise length decrease with 12 assembly steps, with an average decrease of 31±14 nm. This is about half of the 56 nm found for the decrease in end-to-end length per linking number unit (Figure 5), suggesting the assembly of 6 nucleosomes. The number of formed supercoils can independently be deduced from the change in linking number (Figure 3B) which yields 7±2 nucleosomes. Finally, during disassembly of the nucleosomes, a total of 13 steps is counted (Figure 3C), providing an estimate of 6.5 nucleosomes. The step counting methods in principle provide a lower limit for the number since it is possible that one misses 1 or a few steps in the measurement. Assuming a 2-step assembly process, the example molecule shown in Figure 3 thus shows an excellent consistency in the three estimates for the number of 7 nucleosomes.

Also for larger numbers of assembly and disassembly steps, the numbers are quite well comparable, as shown in the assembly and disassembly steps in Figures 2H and 6B (79 and 67 steps, respectively). In literature, disruption events have already been described as 2-step processes [16]. The comparable numbers for assembly and disassembly steps are therefore already directly suggestive for a 2-step assembly process. Further support is provided by the size of the assembly steps as each assembly step size is about half the length by which the DNA end-to-end length decreases per formed nucleosome.

Summing up: based on 1) the decrease in end-to-end length per formed positive supercoil which is roughly twice the assembly step size; 2) the number of formed supercoils which is half of the number of assembly steps,12; 3) the comparable number of assembly end disassembly steps combined with the reported 2-step disassembly [16], we conclude that formation of each nucleosome occurs in two separate steps.

### Conclusions and outlook

In summary, we studied nucleosome assembly mediated by the chaperone NAP1 at a single-molecule level in real time with magnetic tweezers. We showed that the number of assembled nucleosomes on each of the DNA molecules can be estimated based on the change in supercoiling density and end-to-end length. Furthermore, we demonstrated that this number can be verified by observing the number of disassembly steps when applying high force. With our experimental conditions, we assembled up to ∼27 nucleosomes on 8 kb long dsDNA within ∼300 seconds. Once the nucleosomes are formed, they are stable, and no disassembly was measured during control experiments of several hours. The data indicate that NAP1-assisted assembly of complete nucleosomes occurs as a two-step process. We suggest that first histones H3 and H4 are associated to the DNA as oligomers, where the DNA is partially wrapped around this substructure, inducing an end-to-end length decrease but no change in linking number. Subsequently, NAP1 brings histones H2A and H2B to the DNA upon which the complete octamer is formed, and the DNA fully wraps around the histone complex, forming a complete nucleosome and changing the linking number.

Importantly, our method allows measuring the number of assembled nucleosomes without disrupting them. Also, all our experiments have been carried out at near-natural conditions where the applied force and torque resembles those in the nucleus and with natural DNA instead of tandem repeats of the often used ‘601’ strong positioning sequence. In addition, compared to the preassembly of nucleosome fibers by salt dialysis, our method provides the possibility of measuring on the same DNA molecule repeatedly and the possibility to test the integrity of the molecules before the addition of the nucleosomes, which is strongly advantageous. Our method thus is well suited to future studies of, for example, chromatin remodeling at the single molecule level.

## Supporting Information

Figure S1 Force-distance curve of a DNA molecule in the magnetic tweezers. This figure shows the relation between the 8 kb dsDNA end-to-end length as a function of the applied force. By fitting the force-distance data to a worm-like-chain model [22], the persistence length, the stiffness of the DNA molecule, of each molecule is calculated. Experiments are continued if the value is close to the expected 50 nm.
(TIF)

Figure S2 Step histogram of the NAP1 assisted assembly of histones H3 and H4. The fitted steps of several protein flushes of 8 different molecules are analyzed with the step-finder algorithm and the result is shown in the histogram. The most likely step size is around −25 nm. The larger steps could be two steps occurring around the same time, and therefore not being recognized as two separate steps. This step size histogram is comparable to figure 2H, which shows the step sizes of NAP1 assisted assembly of all four core histones.
(TIF)
