# Supplementary figures and images for: NAP1-Assisted Nucleosome Assembly on DNA Measured in Real Time by Single-Molecule Magnetic Tweezers
Source: PLoS One. 2012 Sep 25;7(9):e46306. doi: 10.1371/journal.pone.0046306 (PMC3457989; doi:10.1371/journal.pone.0046306)

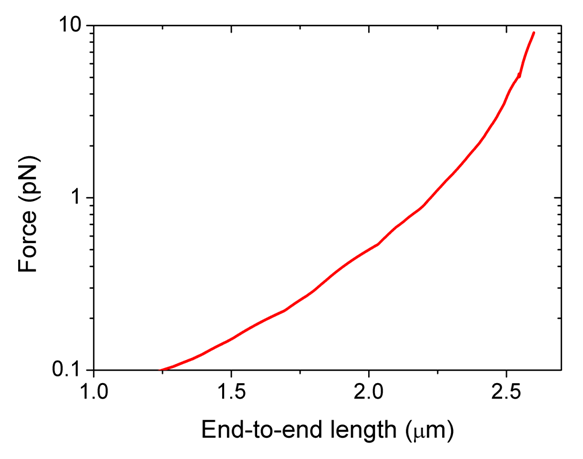

Supplement: Figure S1 — Force-distance curve of a DNA molecule in the magnetic tweezers. This figure shows the relation between the 8 kb dsDNA end-to-end length as a function of the applied force. By fitting the force-distance data to a worm-like-chain model [22], the persistence length, the stiffness of the DNA molecule, of each molecule is calculated. Experiments are continued if the value is close to the expected 50 nm. (TIF) [file pone.0046306.s001.tif]

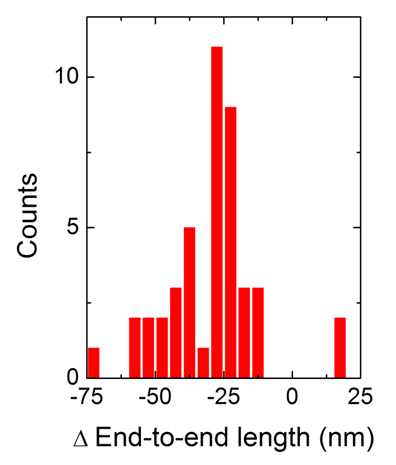

Supplement: Figure S2 — Step histogram of the NAP1 assisted assembly of histones H3 and H4. The fitted steps of several protein flushes of 8 different molecules are analyzed with the step-finder algorithm and the result is shown in the histogram. The most likely step size is around −25 nm. The larger steps could be two steps occurring around the same time, and therefore not being recognized as two separate steps. This step size histogram is comparable to figure 2H, which shows the step sizes of NAP1 assisted assembly of all four core histones. (TIF) [file pone.0046306.s002.tif]
